# A *Streptococcus pneumoniae* lineage usually associated with pneumococcal conjugate vaccine (PCV) serotypes is the most common cause of serotype 35B invasive disease in South Africa, following routine use of PCV

**DOI:** 10.1099/mgen.0.000746

**Published:** 2022-04-06

**Authors:** Kedibone M. Ndlangisa, Mignon du Plessis, Stephanie Lo, Linda de Gouveia, Chrispin Chaguza, Martin Antonio, Brenda Kwambana-Adams, Jennifer Cornick, Dean B. Everett, Ron Dagan, Paulina A. Hawkins, Bernard Beall, Alejandra Corso, Samanta Cristine Grassi Almeida, Theresa J. Ochoa, Stephen Obaro, Sadia Shakoor, Eric S. Donkor, Rebecca A. Gladstone, Pak Leung Ho, Metka Paragi, Sanjay Doiphode, Somporn Srifuengfung, Rebecca Ford, Jennifer Moïsi, Samir K. Saha, Godfrey Bigogo, Betuel Sigauque, Özgen Köseoglu Eser, Naima Elmdaghri, Leonid Titov, Paul Turner, K. L. Ravi Kumar, Rama Kandasamy, Ekaterina Egorova, Margaret IP, Robert F. Breiman, Keith P. Klugman, Lesley McGee, Stephen D. Bentley, Anne von Gottberg, The Global Pneumococcal Sequencing Consortium

**Affiliations:** ^1^​ National Institute for Communicable Diseases (NICD), a division of the National Health Laboratory Service, Johannesburg, South Africa; ^2^​ School of Pathology, University of the Witwatersrand, Johannesburg, South Africa; ^3^​ Parasites and Microbes, Wellcome Sanger Institute, Hinxton, UK; ^4^​ WHO Collaborating Centre for New Vaccines Surveillance, Medical Research Council Unit, The Gambia at London School of Hygiene & Tropical Medicine, Fajara, The Gambia; ^5^​ NIHR Global Health Research Unit on Mucosal Pathogens, Division of Infection and Immunity, University College London, London, UK; ^6^​ West Africa Partnerships and Strategies, Medical Research Council Unit The Gambia at The London School of Hygiene and Tropical Medicine, Fajara, Gambia; ^7^​ Malawi-Liverpool-Wellcome-Trust, Blantyre, Malawi; ^8^​ Centre for Inflammation Research, Queens Research Institute, University of Edinburgh, Edinburgh, UK; ^9^​ The Faculty of Health Sciences, Ben-Gurion University of the Negev, Beer-Sheva, Israel; ^10^​ Rollins School Public Health, Emory University, Atlanta, USA; ^11^​ Centers for Disease Control and Prevention, Atlanta, USA; ^12^​ Administración Nacional de Laboratorios e Institutos de Salud, Buenos Aires, Argentina; ^13^​ Center of Bacteriology, Adolfo Lutz Institute, São Paulo, Brazil; ^14^​ Instituto de Medicina Tropical, Universidad Peruana Cayetano Heredia, Lima, Peru; ^15^​ University of Nebraska Medical Center, Omaha, USA; ^16^​ The Aga Khan University, Karachi, Pakistan; ^17^​ Department of Medical Microbiology, University of Ghana Medical School, Accra, Ghana; ^18^​ Department of Microbiology and Carol Yu Centre for Infection, The University of Hong Kong, Queen Mary Hospital, Hong Kong, PR China; ^19^​ National Laboratory of Health, Environment and Food, Ljubljana, Slovenia; ^20^​ Hamad Medical Corporation, Doha, Qatar; ^21^​ Faculty of Pharmacy, Siam University, Bangkok, Thailand; ^22^​ Papua New Guinea Institute of Medical Research, Goroka, Papua New Guinea; ^23^​ Agence de Médecine Préventive, Paris, France; ^24^​ Child Health Research Foundation, Dhaka, Bangladesh; ^25^​ Kenya Medical Research Institute, Kisumu, Kenya; ^26^​ Centro de Investigação em Saúde da Manhiça, Maputo, Moçambique; ^27^​ Hacettepe University Faculty of Medicine, Department of Medical Microbiology, Ankara, Turkey; ^28^​ Faculty of Medicine and Pharmacy & Ibn Rochd University Hospital Center, Casablanca, Morocco; ^29^​ The Republican Research and Practical Center for Epidemiology and Microbiology, Minsk, Belarus; ^30^​ Centre for Tropical Medicine and Global Health, Nuffield Department of Medicine, University of Oxford, Oxford, UK; ^31^​ Kempegowda Institute of Medical Sciences Hospital & Research Center, Bangalore, India; ^32^​ University of Oxford, and the NIHR Oxford Biomedical Research Centre, Oxford, UK; ^33^​ G. N. Gabrichevsky Research Institute for Epidemiology and Microbiology, Moscow, Russia; ^34^​ Department of Microbiology, Chinese University of Hong Kong, Hong Kong, PR China; ^35^​ The Emory Global Health Institute, Atlanta, USA; ^36^​ Hubert Department of Global Health, Rollins School of Public Health, and Division of Infectious Diseases, School of Medicine, Emory University, Atlanta, GA, USA; ^37^​ The Global Pneumococcal Sequencing Project (http://www.pneumogen.net/gps/.), UK

**Keywords:** South Africa, *Streptococcus pneumoniae*, serotype 35B, global pneumococcal sequence cluster

## Abstract

Pneumococcal serotype 35B is an important non-conjugate vaccine (non-PCV) serotype. Its continued emergence, post-PCV7 in the USA, was associated with expansion of a pre-existing 35B clone (clonal complex [CC] 558) along with post-PCV13 emergence of a non-35B clone previously associated with PCV serotypes (CC156). This study describes lineages circulating among 35B isolates in South Africa before and after PCV introduction. We also compared 35B isolates belonging to a predominant 35B lineage in South Africa (GPSC5), with isolates belonging to the same lineage in other parts of the world. Serotype 35B isolates that caused invasive pneumococcal disease in South Africa in 2005–2014 were characterized by whole-genome sequencing (WGS). Multi-locus sequence types and global pneumococcal sequence clusters (GPSCs) were derived from WGS data of 63 35B isolates obtained in 2005–2014. A total of 262 isolates that belong to GPSC5 (115 isolates from South Africa and 147 from other countries) that were sequenced as part of the global pneumococcal sequencing (GPS) project were included for comparison. Serotype 35B isolates from South Africa were differentiated into seven GPSCs and GPSC5 was most common (49 %, 31/63). While 35B was the most common serotype among GPSC5/CC172 isolates in South Africa during the PCV13 period (66 %, 29/44), 23F was the most common serotype during both the pre-PCV (80 %, 37/46) and PCV7 period (32 %, 8/25). Serotype 35B represented 15 % (40/262) of GPSC5 isolates within the global GPS database and 75 % (31/40) were from South Africa. The predominance of the GPSC5 lineage within non-vaccine serotype 35B, is possibly unique to South Africa and warrants further molecular surveillance of pneumococci.

## Data Summary

**Impact Statement**

Increases in non-PCV serotypes due to expansion of pre-existing clones and emergence of capsule-switch strains have been reported in some countries following the introduction of PCV. In this study, we describe the epidemiology of non-vaccine serotype 35B in South Africa before and after the introduction of PCV. This serotype has become one of the emerging non-PCV serotypes in South Africa following PCV introduction and its dominance appears to be driven by a pre-existing lineage (GPSC5/CC172), which, in South Africa, was previously dominated by PCV serotype 23F prior to the introduction of PCV. These findings contribute to the general understanding of pneumococcal molecular epidemiology in the PCV era as well as the international effort to characterize replacement serotypes.

Raw fastq data, assemblies and annotations for 262 samples that were sequenced as part of the Global Pneumococcal Sequencing project (GPS) were previously released [1] to the European Nucleotide Archive. Genome assembles of the 22 serotype 35B isolates from South Africa that were not part of the GPS project has been deposited at GenBank under the BioProject PRJNA339372. Individual accessions for the samples are listed in Table S1 (available in the online version of this article) and are available at Figshare: https://doi.org/10.6084/m9.figshare.16621984 [2].

## Introduction

Pneumococcal conjugate vaccines (PCV) have significantly reduced vaccine-serotype invasive pneumococcal disease (IPD) among vaccinated children in countries where PCV has been introduced [3–5]. Reduction of disease in unvaccinated children and adults because of herd effect has also been reported [3]. Despite the decrease, increases in IPD rates due to some non-PCV serotypes (serotype replacement) were observed in some countries following routine use of PCV [3–7]. Increases in non-PCV serotypes have been attributed to expansion of pre-existing clones; however, capsule-switch strains have also emerged that originated through replacement of capsular locus genes [8–11].

Serotype 35B is an important emerging non-vaccine serotype. Olarte *et al.* and Chochua *et al.* [12, 13] reported an increase in this serotype in the USA due to expansion of a pre-existing antibiotic-resistant clone CC558 (GPSC59), in addition to emergence of antibiotic-resistant CC156 (GPSC6), previously associated with PCV serotypes in the USA. Two different capsular switch events were described in the USA, occurring between 35B/CC558 donor strains and 9V/ST156 recipients, resulting in 35B/CC156 progeny [13, 14].

In South Africa, PCV7 was introduced in 2009 with a three-dose schedule at 6, 14 and 36 weeks of age and was replaced with PCV13 in 2011 using the same schedule. By 2012 (compared to 2005 through 2008), among children less than 2 years of age, the incidence of IPD due to PCV7 serotypes decreased by 89 % and additional PCV13 serotypes, not in PCV7, by 57 % [15]. In 2016, IPD rates of non-PCV (non-PCV13) serotypes among children <5 years of age increased 29 % (4.3 to 5.6 per 100 000 population) compared to 2005–2008 with non-PCV serotypes 8 and 35B as the most causes of IPD [16]. Serotype 35B IPD in children <5 years of age increased 384 % (0.08 to 0.4 per 100 000 population) in 2016 compared to 2005–2008.

In our previous study describing baseline genetic structure within pneumococcal serotypes in 2007, prior to routine PCV use in South Africa, we found that predominant sequence types circulating among some serotypes differed from predominant sequence types identified globally [17]. Molecular surveillance of isolates from South Africa, particularly for emerging non-vaccine serotypes such as 35B, is therefore important as their emergence could be due to different lineages than those described elsewhere, and might be a sentinel for further geographic spread of this serotype. The aim of this study was to describe and compare lineages circulating among 35B isolates in South Africa before and after PCV introduction, and to compare isolates belonging to the predominant 35B lineage circulating in South Africa to those in other parts of the world.

## Methods

### National IPD surveillance

Isolates were collected as part of GERMS-SA, a national, laboratory-based surveillance programme for IPD in all nine provinces in South Africa, initiated in 1999 [18]. In 2003, systematic collection of patient clinical data including outcome and HIV serological status from approximately 30 sentinel sites in all nine provinces commenced. Isolates and patient data for this study were from IPD cases reported from 2005 through 2014, and were submitted to the reference laboratory at the National Institute for Communicable Diseases (NICD). A case of IPD was defined as the isolation of *S. pneumoniae* from a normally sterile-site specimen (e.g. blood, cerebrospinal fluid [CSF], pleural fluid, joint fluid). In addition, for invasive disease rate calculations, cases included patients with normally sterile-site specimens testing positive by PCR [19], or bacterial latex antigen supported by Gram-stain microscopy.

### Serotyping and antimicrobial susceptibility testing

Serotypes of pneumococcal isolates were determined by the Quellung method using serotype-specific antisera (Statens Serum Institute, Copenhagen, Denmark) [20]. Isolates were phenotypically classified as serotype 35B if they bound to pool G, group 35 antiserum and factor sera 35 a, 35 c and 29b. Antimicrobial MIC testing was performed by agar dilution (for penicillin and ceftriaxone) or Etest (amoxicillin, erythromycin, clindamycin, chloramphenicol, tetracycline, rifampicin, cotrimoxazole, ofloxacin, linezolid and vancomycin) (AB Biodisk, Solna, Sweden) from 2005 through 2008. From 2009 to 2014, commercially prepared Sensititre-SASP2 panels (Trek Diagnostics, Cleveland, OH) were used for broth microdilution. Results were interpreted according to the 2014 Clinical and Laboratory Standards Institute (CLSI) guidelines and breakpoints [21]. Isolates with penicillin MICs ≥0.12 mg l^−1^ were considered non-susceptible to penicillin. For other antimicrobials, isolates were defined as non-susceptible if they were intermediately or fully resistant to the agent tested, according to CLSI guidelines. Multidrug resistance was defined as non-susceptibility to beta-lactams and at least two other classes of antimicrobials.

### Genetic characterization

A subset of isolates collected through our national IPD surveillance from South Africa in 2005 to 2014 were whole-genome sequenced as part of the Global Pneumococcal Sequence project (GPS) (http://www.pneumogen.net/gps/). The sampling strategy for the GPS project was as follows: 300 isolates per year, representing all serotypes and from patients of all ages in South Africa, were selected. Selection was random with respect to serotype and was stratified by age for each year: 150 isolates from children aged 0 to 2 years, 75 isolates from children aged 3 to 5 years and 75 isolates from individuals >5 years of age. As part of the GPS project, only 45 of the 262 isolates that were phenotypically determined to be serotype 35B were selected. We therefore randomly selected an additional 22 35B IPD isolates submitted during 2005 to 2008 (pre-PCV period) to increase the number of isolates for this study.

DNA was extracted from the 67 35B isolates using the QIAamp DNA mini kit (Qiagen, Venlo, Netherlands) and DNA extracts were quantified using the Qubit instrument and dsDNA BR Assay kit (Life Technologies, Carlsbad, CA, USA). Multiplexed paired-end libraries were prepared using the Nextera XT DNA sample preparation kit (Illumina, San Diego, CA, USA). Genome sequencing was carried out on an Illumina MiSeq platform.

Multi-locus sequence types (clonal complexes and sequence types), Global Pneumococcal Sequence Clusters (GPSCs), *in silico* serotypes, and resistant genotypes to penicillin, tetracycline, erythromycin, chloramphenicol and co-trimoxazole were derived from WGS data, as previously described [1, 14]. *In silico* serotype was determined from genome data using PneumoCaT and SeroBA [22]. Penicillin-binding protein (PBP) profiles were assigned based on transpeptidase domain amino acid sequences of 277–359 residues from PBPs 1a, 2b and 2x with three-number combination PBP genes referred to with standard nomenclature (pbp1a-pbp2b-pbp2x) [14]. A clonal complex was defined as a group of related STs sharing six of seven identical alleles with another ST in the group.

In addition to the 35B isolates from South Africa the study included 262 *S. pneumoniae* genomes representing GPSC5 : 115 from South Africa and 147 from other parts of the world that were sequenced as part of the larger GPS project [1]. GPSC5 is a predominant lineage within 35B in South Africa.

### Statistical analysis

Average incidence for 2005–2008 (pre-PCV period), 2009–2010 (PCV7 period) and 2011–2014 (PCV13 period) was calculated by dividing the average number of cases by mid-year population estimates data from Statistics South Africa [23] and multiplying the quotient by 100 000. Differences during the pre-PCV era, PCV7 period and PCV13 period, in prevalence and antimicrobial susceptibility of isolates belonging to clonal complexes/GPSCs were assessed using univariate analysis using Stata version 14 (StataCorp, College Station, USA).

## Results

### Invasive pneumococcal disease surveillance, South Africa

**Fig. 1.**
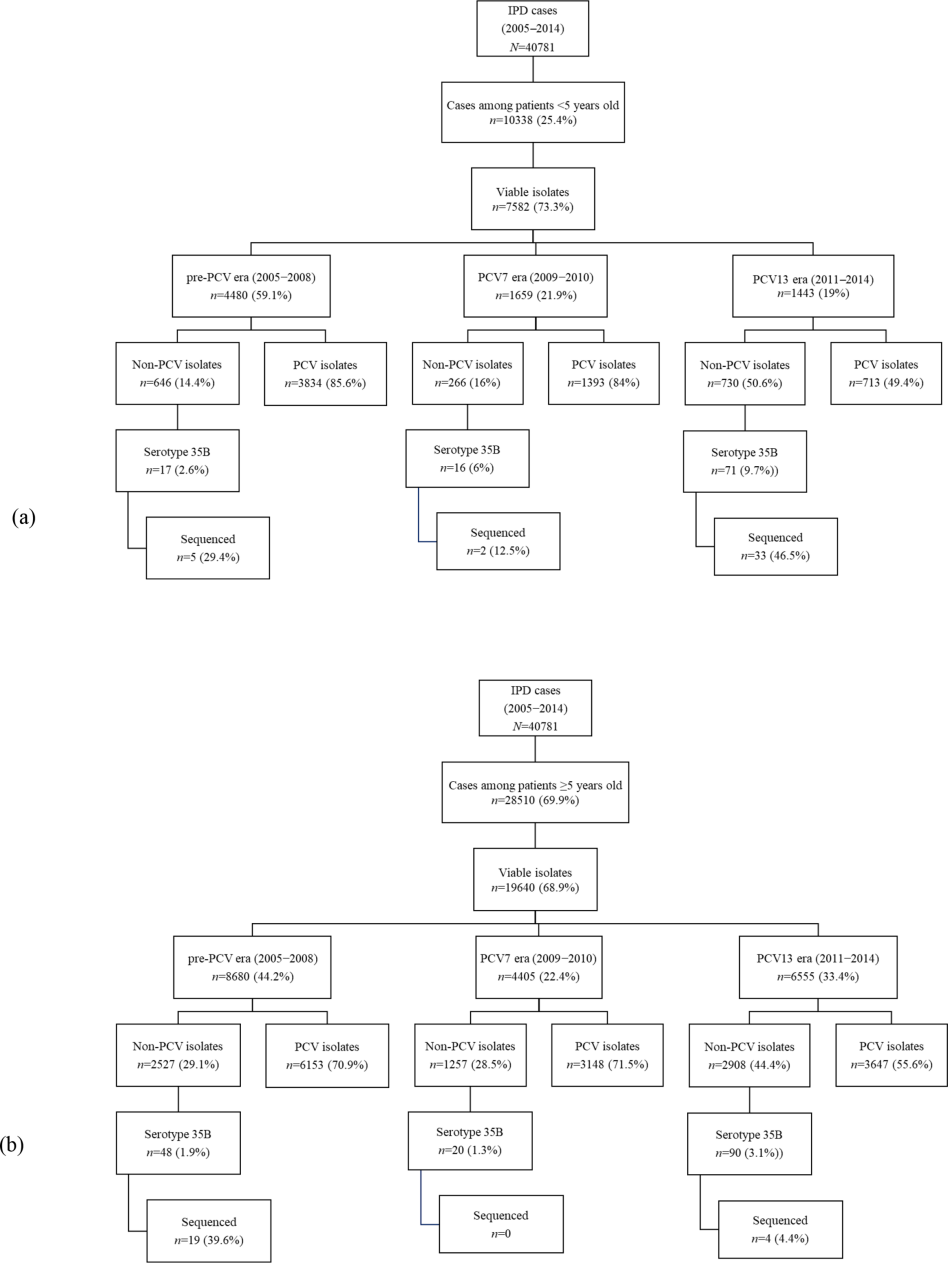
Invasive pneumococcal disease cases and isolates obtained from 2005 through 2014 among South African (a) children <5 years and (b) individuals ≥5 years old.

**Fig. 2.**
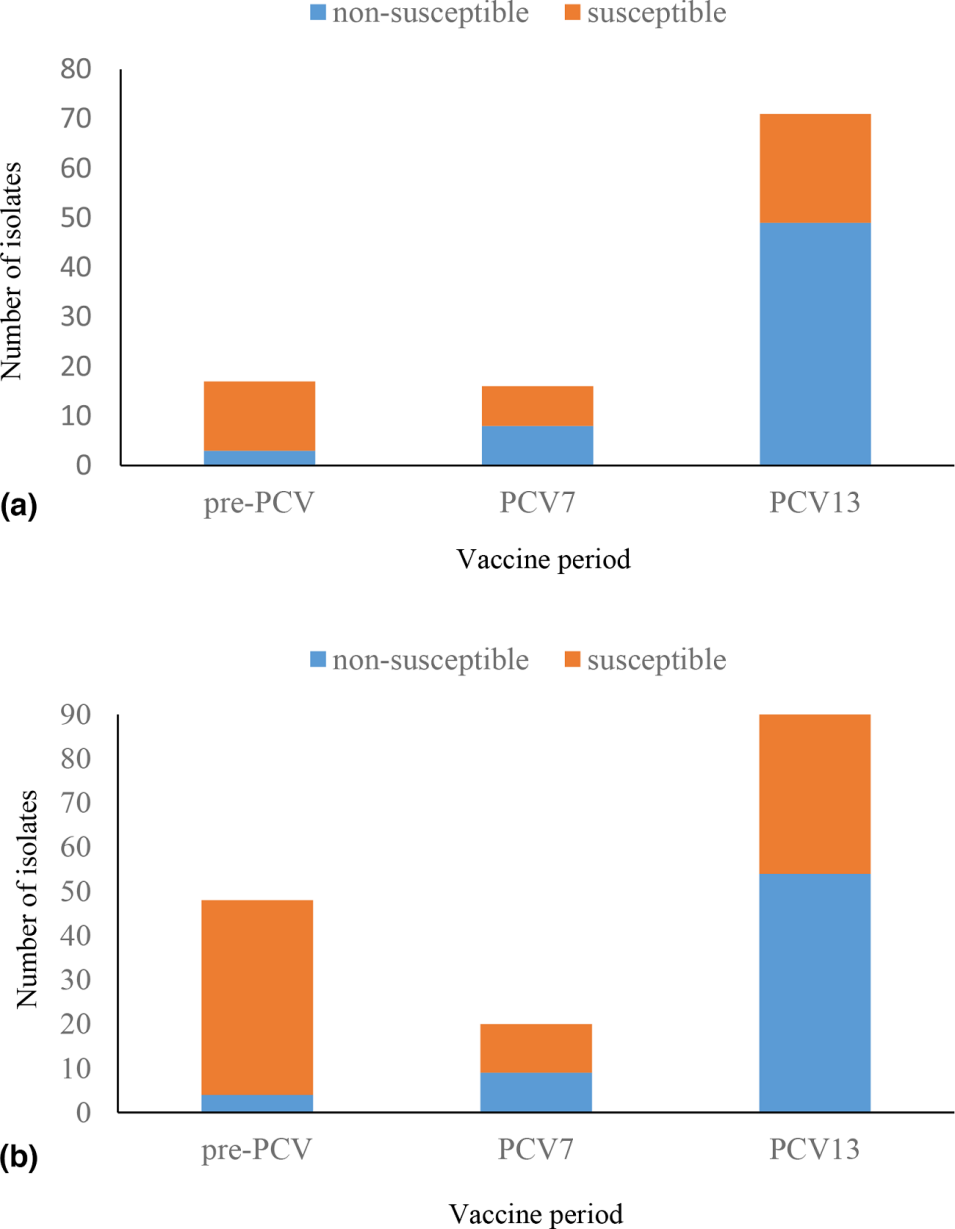
Distribution of penicillin non-susceptible and susceptible serotype 35B pneumococcal isolates causing invasive disease in South Africa among (a) children <5 years old (*N*=104) during the pre-PCV (2005–2008) (*n*=17), PCV7 (2009–2010) (*n*=16) and PCV13 (2011–2014) period (*n*=71) and (b) patients ≥5 years old (*N*=158) during the pre-PCV (2005–2008) (*n*=48), PCV7 (2009–2010) (*n*=20) and PCV13 (2011–2014) period (*n*=90).

During the study period, 40 781 IPD cases were reported of which 28 229 (69.2 %) had viable isolates available for further characterization. Age was known for 27 224 (96.4 %) cases with viable isolates, of which 7582 (27.9 %) were from children <5 years old (Fig. 1). Within this age group, 0.4 % (17/4480), 1 % (16/1659) and 4.9 % (71/1443) of isolates obtained during the pre-PCV, PCV7 and PCV13 periods, respectively, were phenotypically identified as serotype 35B. Among individuals aged ≥5 years, serotype 35B represented 0.6 % (48/8680), 0.5 % (20/4405) and 1.3 % (90/6555) of isolates during the pre-PCV, PCV7 and PCV13 periods, respectively. In total, 262 serotype 35B isolates were phenotypically identified among IPD isolates during the study period, from individuals of all ages in South Africa. Among children <5 years old the average incidence of serotype 35B increased by 290 % (95 % confidence interval [CI], 286% to 1485%) from 0.08 cases per 100 000 person-years in the pre-PCV era to 0.3 cases per 100 000 person-years during the PCV13 period. Among patients aged ≥5 years, the rate of serotype 35B IPD did not change (0.03 vs. 0.05 per 100 000 person-years pre-PCV vs. PCV13 era). The proportion of penicillin non-susceptible serotype 35B isolates increased from 17.6 % (3/17) during the pre-PCV era to 69 % (49/71) during the PCV13 era among children <5 years old (*P*<0.001), and from 8.3 % (4/48) to 60 % (54/90) among patients ≥5 years old (*P*<0.001) (Fig. 2).

### Serotype 35B genotypes

**Fig. 3.**
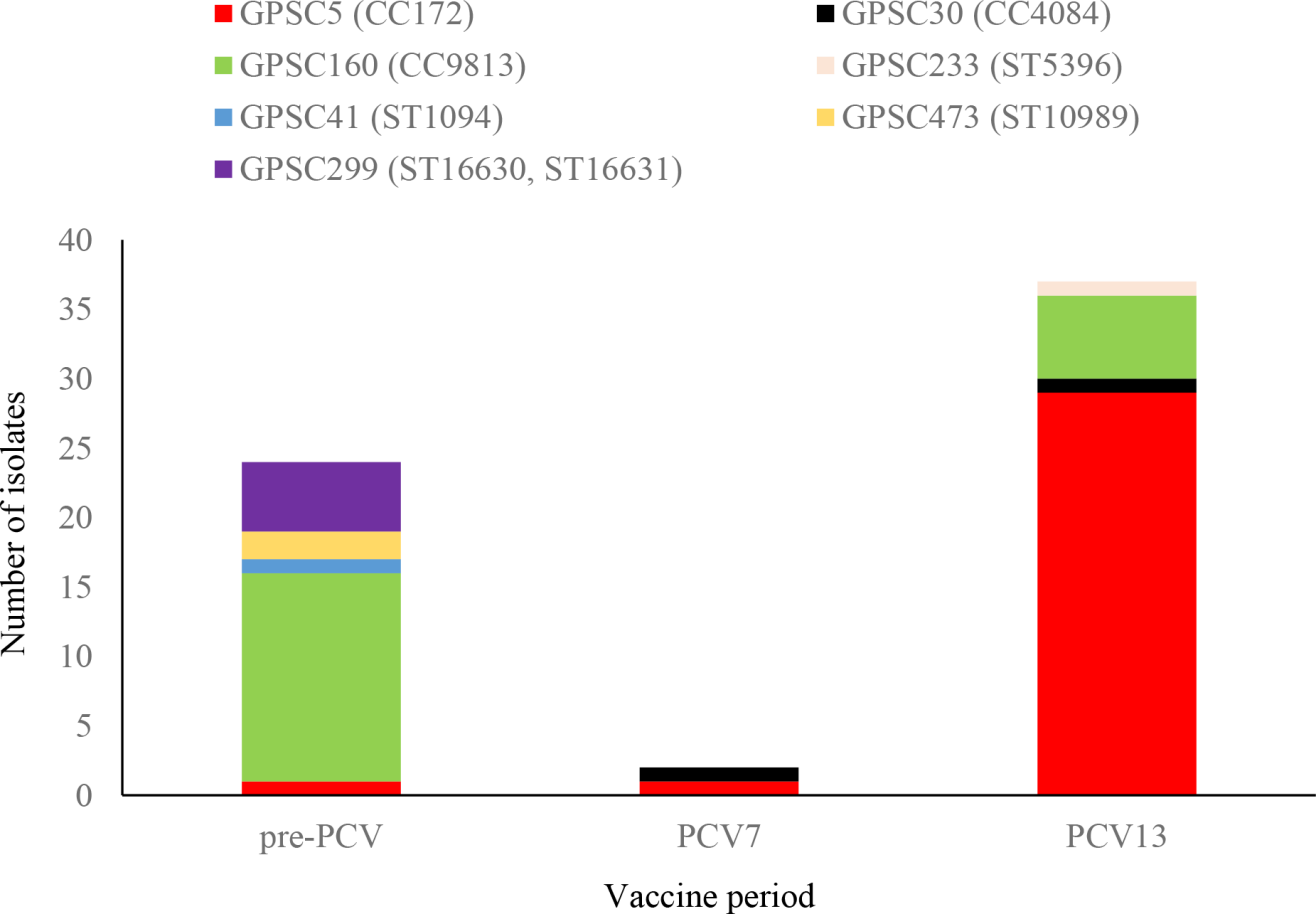
Distribution of global pneumococcal sequence clusters (GPSCs), clonal complexes (CC) and sequence types (STs) among 63 serotype 35B isolates causing invasive disease in South Africa during the pre-PCV (2005–2008) (*n*=24), PCV7 (2009–2010) (*n*=2) and PCV13 (2011–2014) period (*n*=37).

Sixty-seven (26 %) of the 262 isolates that were phenotypically identified as 35B were sequenced. *In silico* serotype assignment confirmed 63 of the 67 sequenced isolates to be serotype 35B (four isolates were identified as serotype 35D and were excluded from this analysis). The majority of 35B isolates were from children <5 years old (40/63, 63 %). The 35B isolates were differentiated into seven GPSCs and belonged to three clonal complexes, namely CC172, CC9813 and CC4084, and five unrelated sequence types (Fig. 3). GPSC5 was the most common lineage and represented 49 % (31/63) of the isolates, all of which were CC172. GPSC160 represented 33 % (21/63) of the isolates, all of which were CC9813. Two isolates belonged to GPSC30 and were both CC4084. The remaining nine isolates belonged to four different GPSCs and five sequence types.

The majority of sequenced 35B isolates were from the PCV13 period (61 %, 37/63). During this period, GPSC5 was represented by 78 % (29/37) of the isolates, GPSC160 by 16 % (6/37); GPSC30 and GPSC233 by one isolate each. Two isolates from the PCV7 era were sequenced, one belonged to GPSC5 and the other to GPSC30. Among 24 isolates from the pre-PCV period that were sequenced, GPSC160 was the most common cluster (63 %, 15/24). One isolate belonged to GPSC5 (CC172) and the remaining eight isolates belonged to three different GPSCs.

### GPSC5 representation among all serotypes in South Africa

**Fig. 4.**
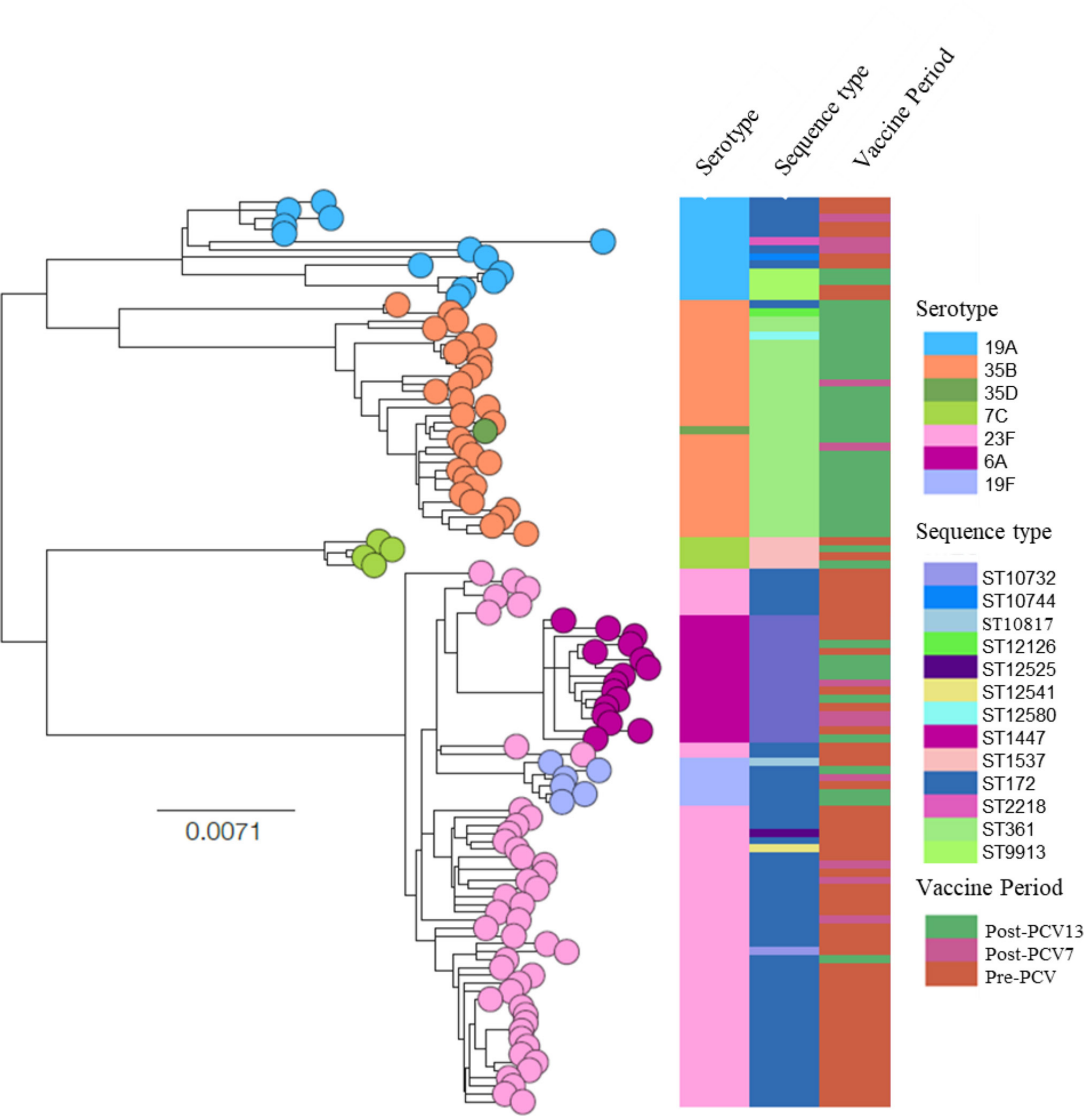
Phylogeny of GPSC5 (CC172) isolates causing invasive disease in South Africa (*N*=115) during the pre-PCV (2005–2008) (*n*=46), PCV7 (2009–2010) (*n*=25), and PCV13 (2011–2014) (*n*=44) period. Leaf nodes on the tree are colour coded according to serotype. Microreact: https://microreact.org/project/M-plmPZP5.

Among ~5000 IPD isolates from South Africa, from 2005 through 2014, that were sequenced, 115 isolates belonged to GPSC5 (Fig. 4). The isolates expressed one of seven serotypes, namely, 6A, 7C, 19A, 19F, 23F, 35B or 35D. While serotype 23F was the most common serotype among GPSC5 isolates during both the pre-PCV (80 %, 37/46) and PCV7 periods (32 %, 8/25), only 2 % (1/44) of GPSC5 isolates were serotype 23F during the PCV13 period. During the PCV13 period, serotype 35B was the most common serotype among GPSC5 isolates (66 %, 29/44).

### GPSC5 antibiotic resistance and penicillin-binding protein (*pbp*) profiles

Almost all (97 %, 111/115) GPSC5 isolates from South Africa were non-susceptible to penicillin (MIC_50_ : 0.25 µg ml^−1^ and MIC_90_ : 1 µg ml^−1^) and 18 %(21/115) were multi-drug resistant. All serotype 35B isolates (*n*=31) belonging to GPSC5 were non-susceptible to penicillin; none were multi-drug resistant. The serotype 35B GPSC5 isolates were classified into two *pbp* profiles (7-1-455 and 7-1-242). The majority of serotype 35B isolates (90 %, 29/31) were 7-1-455. Neither profile was identified among non-35B GPSC5 isolates.

### GPSC5 representation among the GPS global dataset

**Fig. 5.**
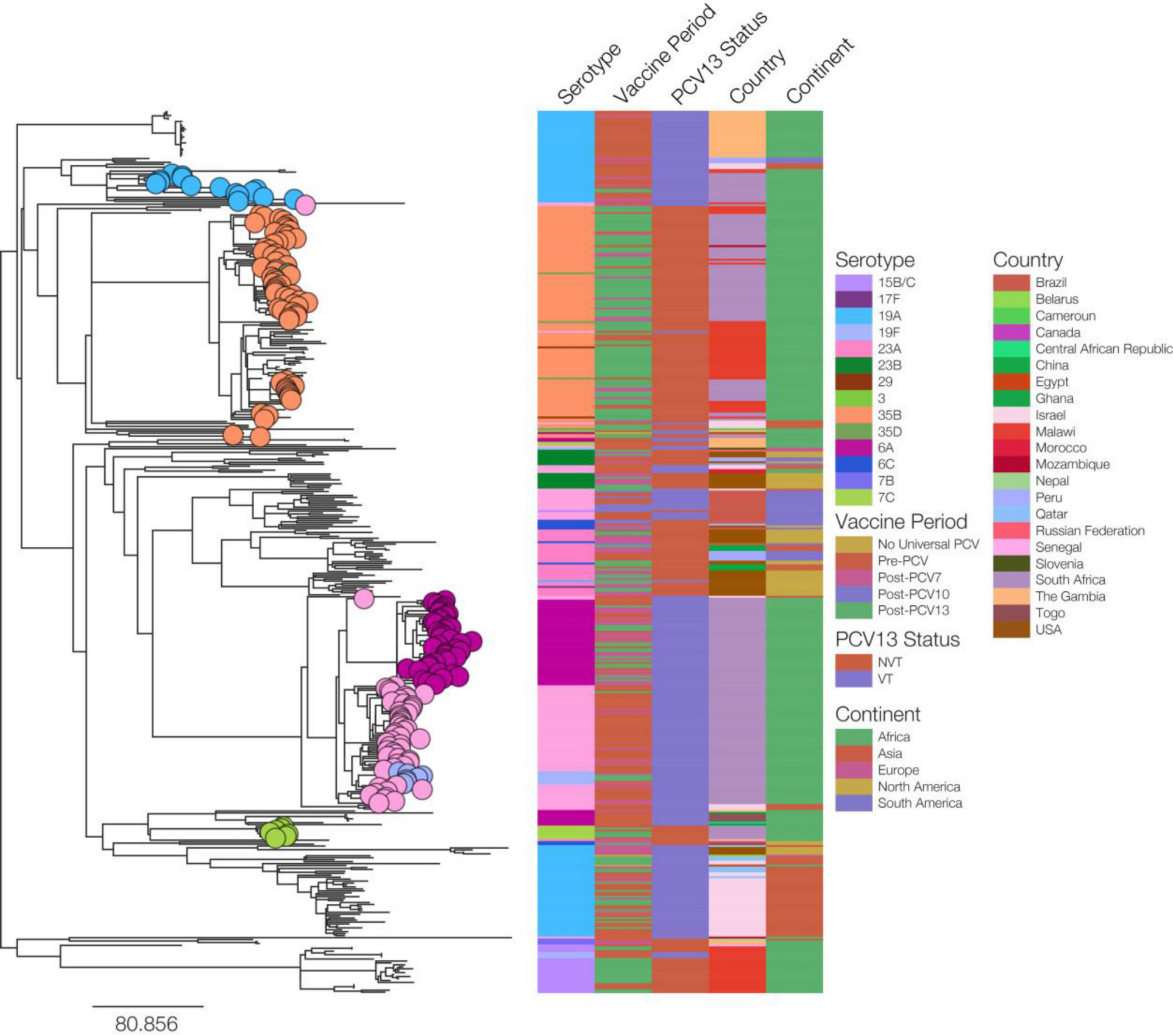
Phylogeny of GPSC5 isolates (*N*=262) causing pneumococcal disease globally, 2005–2014. Leaf nodes on the tree are colour coded according to serotype, only nodes for isolates from South Africa are shown.

A total of 262 isolates belonging to GPSC5 were identified among IPD isolates in the GPS global dataset, from 2005 through 2014 (Fig. 5). The majority of GPSC5 isolates were from South Africa (44 %, 115/262), followed by Israel (18 %, 48/262), the USA (15 %, 38/262), Brazil (7 %, 19/262) and Malawi (5 %, 12/262). Serotype 35B represented 15 % (40/262) of GPSC5 isolates. The majority of serotype 35B isolates were from South Africa (78 %, 31/40). The remaining nine serotype 35B isolates were from Malawi (*n*=4), Israel (*n*=3), Mozambique (*n*=1) and Bangladesh (*n*=1). The nine serotype 35B isolates not from South Africa were penicillin non-susceptible but none were multi-drug resistant. They were differentiated into three *pbp* profiles [7-1-455 (*n*=5), 7-1-77 (*n*=3) and 7-1-462]. Other than one serotype 23F isolate from Israel that was classified as *pbp* 7-1-77, the three profiles were not identified among other GPSC5 isolates.

## Discussion

In this study we report an increase in the average incidence of serotype 35B IPD among children <5 years old in South Africa during the post-PCV13 period compared to pre-PCV. We identified GPSC5, a lineage usually associated with PCV serotypes, as the most common lineage among the non-PCV serotype 35B isolates in South Africa during the PCV13 period. While PCV serotype 23F was the most dominant serotype among isolates belonging to GPSC5 during the pre-PCV period, serotype 35B became the leading GPSC5/CC172 serotype during the PCV13 period.

Serotype 35B has been shown to be one of the non-PCV replacement serotypes following the implementation of PCV [12, 13, 24]. Demonstration of a high proportion (up to 90 % in some locations) of serotype 35B isolates which are penicillin non-susceptible [24] makes replacement with serotype 35B a particular concern. In South Africa, where the incidence of serotype 35B invasive disease increased among children <5 years old during the post-PCV13 era compared to the pre-PCV era, the proportion of 35B isolates that are penicillin non-susceptible is 80 %.

CC558 is reportedly the main serotype 35B genotype in Japan and in the USA and is associated with multi-drug resistance [13, 24–26]. Following PCV implementation in the USA CC558 has expanded [12, 13, 27]. In South Africa, CC558 (GPSC59) is rare among serotype 35B IPD isolates, instead, CC172 (GPSC5) is the most common lineage during the PCV13 period. Prior to PCV implementation in South Africa GPSC5 was more common among serotype 23F isolates however there was a shift towards serotype 35B as the main GPSC5 serotype during the PCV13 era. This lineage is usually associated with the PCV serotypes 6A, 6B, 23F and 19A [28–30]. The detection of this lineage within isolates of diverse serotypes is in keeping with the higher than average propensity for recombination as measured using r/m previously (GPSC5 r/m 10.16, average 7.70) [1]. A recent analysis of 3233 IPD genomes in the GPS study, from six countries, in children <3 years of age, revealed the expansion of serotype 35B/D within GPSC5 in South Africa [31]. We corroborated this finding in our dataset, which included additional genomes from individuals of all ages. Antimicrobial resistance within this lineage could also be a key contributor to its expansion. Similar to other serotype 35B isolates, penicillin non-susceptibility was high among our 35B isolates belonging to GPSC5, which almost all had the same PBP type. GPSC5, also classified as PMEN26 (or Colombia^23F^-26), is an internationally disseminated penicillin-resistant clone that was first described in serotype 23F isolates in the 1990s (https://www.pneumogen.net/pmen/index.html). The propensity of this lineage to express different capsular types was demonstrated in the USA with the emergence of non-vaccine serotype 23A isolates associated with the 23F global clone, post-PCV7 introduction [32].

Within the global GPS dataset, isolates belonging to GPSC5 were almost exclusively detected (89 %, 232/262) on the African continent and all 35B/35D isolates within this lineage were from Africa [31]. This finding is in agreement with our previous findings [17, 33] that, for some serotypes in South Africa, predominant genotypes are not the same as those circulating in other parts of the world.

Limitations to this study include the fact that not all serotype 35B isolates were sequenced and therefore the serotype 35B genetic population structure described here may not be an accurate reflection of all genotypes circulating in South Africa. Nonetheless, among a collection of over 1000 35B isolates on PubMLST, only seven belong to CC172 [34], supporting our suggestion that the predominance of the GPSC5 lineage within serotype 35B is unique to South Africa and the African continent.

The predominance of GPSC5/CC172, previously associated with serotype 23F in South Africa, among 35B isolates, following PCV introduction in South Africa highlights the importance of molecular surveillance of pneumococci.

## Supplementary Data

Supplementary material 1
